# Changes in Daily Life during the COVID-19 Pandemic among South Korean Older Adults with Chronic Diseases: A Qualitative Study

**DOI:** 10.3390/ijerph18136781

**Published:** 2021-06-24

**Authors:** Juah Kim, Yeonghun Kim, Jiyeon Ha

**Affiliations:** 1 Department of Nursing, Korean Bible University, Seoul 01757, Korea; jooa04@bible.ac.kr; 2 Advanced Institute of Convergence Technology, Suwon 16229, Korea; toya84@snu.ac.kr; 3 College of Nursing, Konyang University, Daejeon 35365, Korea

**Keywords:** COVID-19, lifestyle, chronic disease, aged, qualitative research

## Abstract

Amid the COVID-19 pandemic, older adults are considered a high-risk group and have been advised to stay home or practice social distancing. This qualitative study examined the effects of strong quarantine measures and social distancing on older adults’ lifestyles. The participants in this study were 13 people aged 65 and older with chronic diseases who resided in South Korean communities. Qualitative content analysis was conducted to interpret the data collected from in-depth interviews. Four themes and 13 subthemes were identified. The four themes were “lifestyle changes,” “increased cautiousness in daily life,” “psychological changes,” and “adaptation to life during the COVID-19 pandemic.” The participants followed quarantine rules strictly and noted lifestyle changes, such as increased time spent at home due to social distancing guidelines, a smaller radius of daily activity, and changes in exercise and dietary habits. They also reported increased caution toward other people and objects that other people interacted with due to their fear of COVID-19 infection. They expressed fear about COVID-19 infection and anxiety about COVID-19-related news, and they often felt bored and depressed; however, the participants accepted, endured, and gradually adapted to these lifestyle changes. Non-face-to-face community support is urgently needed for older adults facing reduced levels of physical activity and psychological hardships due to the COVID-19 pandemic.

## 1. Introduction

The coronavirus disease 2019 (COVID-19) pandemic poses a major threat to the health of older adults [1]. Older adults, especially those with underlying diseases or frailty, are a high-risk group for COVID-19 [2,3,4]. Governments around the world have advised older adults to stay at home or maintain social distance from their neighbors to protect them from COVID-19 infection [2,3,5]. Ironically, such social distancing measures themselves constitute another threat to the health of older adults [2,6]. Social isolation may lead to aggravated cardiovascular and autoimmune diseases in older adults [7], as well as worsened symptoms of dementia caused by reduced cognitive stimulation due to fewer social interactions [8]. Moreover, reduced physical activities from limited mobility may lead to physical weakness [6], and the fear, stress, and loneliness resulting from social isolation may negatively affect the overall health of older adults [9].

Approximately 88% to 90% of South Korean older adults in their 60s and 70s have chronic conditions [10], and most are in need of medical services such as regular doctor visits and medication to manage their conditions. COVID-19 quarantine measures, however, have limited the range of possible public activities as a result of social distancing and stay-at-home orders [5,11]. Research in Spain showed that the family members of older adults mainly took care of activities such as grocery shopping and pharmacy visits on behalf of their high-risk relatives during the COVID-19 pandemic [12]. Some seniors have had difficulties accessing medical services during the pandemic [3,13], and some older adults who live alone have faced challenges preparing medications or meals [14]. Many older adults have also reported psychological distress, a negative psychological state, and sleep issues [12,15]. Older adults with multiple chronic diseases are highly vulnerable to physical weakness [16], and may have difficulty self-managing their conditions due to weakened physical functions caused by disease [17]. Therefore, special attention should be paid to older adults’ health during the pandemic, since the use of social resources is limited as a result of social distancing and stay-at-home measures [13].

The results of a study that examined changes in the physical activity behaviors of the older adults during the COVID-19 pandemic suggested that the types and patterns of physical activities that seniors undertake have changed as a result of the pandemic, and that restrictions and anxiety about physical activities caused by social distancing and limited outdoor activities have led some seniors to give up exercising altogether [18]. A decreased level of physical activity has a negative impact on the immune function of older adults [19]. The lifestyles of older adults are more vulnerable to the negative impacts of the pandemic than those of younger adults [20]. Older adults have been deprived of community resources during the pandemic because senior day care centers and public health centers have paused their programs [5]. Continued social distancing may also undermine the physical and mental health of older adults due to increased social isolation and loneliness [3]. 

The COVID-19 pandemic, which has lasted for more than a year, has changed many parts of our lives [21]. This study aimed to explore the impacts of quarantine and social distancing measures on the lifestyle of older adults with chronic diseases living in communities in South Korea. Understanding the experiences of older adults who require management for chronic conditions will have positive implications for the development of nursing services and health promotion programs tailored to seniors during the COVID-19 pandemic.

## 2. Materials and Methods

### 2.1. Study Design

This is a qualitative study with the purpose of developing an in-depth understanding of the changes in the lives of community-dwelling older adults with chronic diseases during the COVID-19 pandemic.

### 2.2. Participants

The participants of this study were Korean residents of three communities in Seoul, Daejeon, and Chungcheong Province aged 65 and older with one or more chronic diseases. Participants understood the purpose of the study and voluntarily agreed to participate. Those who did not have any chronic conditions or who had been diagnosed with a cognitive disability were excluded from the study. Participants were first purposively sampled by soliciting recommendations from senior welfare center staff in each community who were asked to give the names of people living in their communities who they thought would be able to respond truthfully and expressively when asked about their lifestyle changes during the pandemic. Additional participants were recruited using the snowball sampling method by contacting further potential participants who were referred by the purposively sampled participants. Data collection was carried out until data saturation was reached, which was considered to have occurred once interviews stopped yielding new information compared to the analysis of previous interviews. The final number of participants was 13 people. Among them, 10 (76.9%) were women, and the ages of participants ranged from 65 to 80 (mean age: 73.69). Participants were most often high-school graduates (*n* = 6) and resided in Seoul (*n* = 8). In total, 46.2% (*n* = 6) lived alone, and each participant had 1 to 7 chronic diseases (mean: 2.62). The chronic diseases of the participants included hypertension, diabetes, hyperlipidemia, degenerative arthritis, osteoporosis, gout, asthma, chronic colitis, and chronic prostatic hyperplasia. No participants reported psychiatric disorders such as depression. Ten (76.9%) participants were working, and a majority (*n* = 10) of participants said that their subjective socioeconomic status was “low” (Table 1). 

### 2.3. Interviews and Procedures

#### 2.3.1. Research Team

The lead authors on the team have actively conducted research on older adults in South Korea and have attended a variety of seminars and workshops in the overall qualitative research process as members of the Korean Association for Qualitative Research. They previously collaborated on the publication of a qualitative study on experiences of self-management of multiple chronic diseases among older workers. The researchers held regular meetings to read literature on qualitative research methods and to share opinions on data analysis.

#### 2.3.2. Interviews

Participants were recruited by identifying individuals who attended senior welfare centers using the purposive sampling and snowball sampling methods between November 2020 and February 2021. The two lead authors participated in data collection. Individual in-depth interviews were held in interview rooms and classrooms to ensure a comfortable and quiet environment in which to focus. The interviews were held following COVID-19 guidelines, which included wearing masks, checking temperatures, hand sanitizing, and remaining separated by acrylic screens. Tables and chairs were arranged so that the researcher and the participant faced each other, and recording devices were placed on tables near both individuals to minimize missing interview content. Prior to starting the interview, participants were reminded of the details related to the research, including the purpose, process, and that they were being recorded, and signed voluntary written consent forms. Next, personal information on sex, age, education, residence, household members, type and number of diagnosed chronic diseases, occupation, subjective socioeconomic status, and religion was collected prior to the interview.

The interview started with a non-structured and open question (“What are the impacts of the COVID-19 pandemic on older adults’ health and their lives?”) and moved on to more specific questions on the individual lifestyles of the older adults with chronic diseases and their self-management experience. Researchers encouraged participants to answer the questions as freely as possible, and meaningful non-verbal behaviors were recorded as notes for reference during analysis. Each interview lasted approximately 40 to 60 min. The language of the interviews was Korean. Participants were monitored closely for any dizziness or feeling of sickness during the interview, as well as for any discomfort from wearing a mask. After the analysis of the results was completed, this manuscript was edited by a professional biomedical editor who is a native English speaker.

#### 2.3.3. Transcripts

All interviews were recorded with participants’ consent and were transcribed immediately after the in-depth interviews were completed. The recorded audio files were saved under each participant ID. The files were transcribed by a research assistant exactly as expressed by the participants, and any uncertain content was highlighted and confirmed by the researcher who conducted the interview. The final transcriptions were cross-checked by the researchers who carried out the analyses to ensure the accuracy of the transcription data.

### 2.4. Ethical Considerations

The team began data collection only after receiving approval from the Institutional Review Board of Konyang University (IRB No. KYU-2020-123-01). Participants were notified in detail, both in written form and verbally, about the purpose, process, and content of the study, and were informed of the voluntary nature of participation, confidentiality, and the fact that the interviews would be audio-recorded. The participants were asked to sign voluntary written consent forms for participation in the study and the use of private information if they agreed to and understood the terms of the study after receiving an explanation from the researchers. Additionally, they were told that they could withdraw from participation in the study at any time, and that they would have access to the transcriptions and study outcomes whenever they requested it. Participants were also notified repeatedly that the interview would be recorded prior to the beginning of the interview, and efforts were made to maintain participants’ anonymity as much as possible. For example, recorded voice files and transcriptions were managed using participant IDs, removing personal information that could identify participants, including names. A gift was provided to thank them for their participation.

### 2.5. Analysis

After transcription, the conventional content analysis method was used to explain and describe the phenomenon truthfully, as part of the qualitative content analysis approach [22,23]. Content analysis is a method for systematically and objectively describing phenomena and validating theoretical issues to help understand data [24]. The purpose of qualitative content analysis is to achieve an understanding and knowledge of research phenomena by systematically classifying written or oral data into categories with similar meanings [25]. Initially, researchers read the transcriptions and notes made on-site multiple times to obtain a sense of immersion and a sense of the whole [23,26]. Secondly, significant, meaningful statements were highlighted and coded after thoroughly reviewing the data word-for-word [23]. Third, the codes derived from this step in the process were grouped by similar or relevant units into categories. Fourth, these categories were then grouped into meaningful clusters by theme [27,28]. Fifth, the validity and reliability of the outcomes were ensured by exchanging feedback and opinions among researchers as well as participants to confirm whether the outcomes accurately reflected the essence and the meaning of the study’s theme. Microsoft Excel 2016 (part of the MS Office 2016 suite, Microsoft, Redmond, WA, USA) was used for qualitative data analysis. After the analysis of the results was completed, a bilingual translator performed the primary translation, and secondary editing was completed by the above-mentioned biomedical editor. The researchers then identified discrepancies between Korean and English (target language) through back-translation [29]. If a discrepancy was found, the researchers discussed the wording until agreement was reached.

### 2.6. Rigor

The researchers of this study reviewed the four aspects of Lincoln and Guba’s model of trustworthiness, which includes truth value, applicability, consistency, and neutrality, to enhance the validity and reliability of the results [30]. The audio files were checked for any omissions or distortions immediately after the interview to confirm their truth value, and the transcriptions by a research assistant were cross-checked by researchers to ensure accuracy. One of the participants collaborated in the review work to conduct a member-check for consistency between the content and description of the analysis results with their empirical statements. Considering the differences between the source language (Korean) and the target language (English), a professional translator participated in the translation and editing, and inconsistencies of meaning were identified through back-translation into Korean by researchers. In addition, for triangulation, three researchers from multiple academic fields participated in data collection and analysis to increase the rigor of the research. The researchers discussed the interview and data analysis processes several times to establish consistency and ensure the study’s credibility. To ensure high applicability—an external validity factor of qualitative research—data collection and analysis were carried out until interviews stopped yielding new information that had not yet been found in the analyses of previous interview data, which is when data saturation was determined to have been reached. This was performed to ensure that all of the various impacts that the COVID-19 pandemic had on the lifestyles of older adults were represented so that a variety of different themes could be identified and examined during analysis. The results were also validated in terms of whether they were meaningful and applicable by a non-participant who had the same profile as target participants in this study (aged 65 or older with chronic diseases). The non-participant was recommended by a senior welfare center manager. The researchers tried to set aside their own knowledge, prejudices, or stereotypes through mutual conversations or journals to ensure the neutrality and objectivity of the study, and to ensure that the essence of the experiences of life changes during the COVID-19 pandemic among older adults with chronic diseases was accurately reflected in the results.

## 3. Results

In January 2020, after the outbreak of COVID-19 in Korea, quarantine measures such as wearing masks, social distancing, and banning the use of public facilities were implemented in local areas [18,31]. This study was conducted 10–12 months after the spread of COVID-19, before COVID-19 vaccination became available, and during the social distancing period in Korea.

Four themes, 13 subthemes, and 31 codes were identified resulting from the analysis of data collected from in-depth interviews with 13 participants using the qualitative content analysis method. The four subthemes were lifestyle changes, increased cautiousness in daily life, psychological changes, and adaptation to life during the COVID-19 pandemic (Table 2).

### 3.1. Lifestyle Changes

#### 3.1.1. Following Quarantine Rules in Daily Life

The participants refrained from meeting family members, attending events, or gathering on holidays, in accordance with COVID-19 guidelines. Most of them had engaged in fewer activities in the community since they did not meet their friends as often. The participants tried to follow the guidelines thoroughly by socially distancing from others, wearing masks, and practicing hand hygiene.

*“I still see [my children] sometimes but they don’t visit as often as before.” (Participant 3)*

*“I only stay home except for when I go to church.” (Participant 4)*

*“I sit far away. Sit apart from others. Only two people sit on one bench* *—no more—and the [part-time job] chief tells us to sit apart.” (Participant 6)*

*“I stand apart from others [on the bus]… I am cautious [about contacting others].” (Participant 8)*

*“It used to be difficult and bothersome [to wear a mask], and I often forgot to do it… Now I look for my mask first thing in the morning.” (Participant 11)*

*“I take my personal hygiene seriously. I wash my hands well.” (Participant 13)*

#### 3.1.2. Increased Time Spent at Home

The participants said that their children requested that they avoid going outside and stay home due to concerns about potential COVID-19 infection. Older adults spent more time at home, spending a majority of their time watching TV indoors or looking after grandchildren.

*“I don’t go out a lot. [My children] would not let me.” (Participant 7)*

*“TV is my friend when I’m home. A friend.” (Participant 4)*

*“I’m tired but need to go watch my grandchildren… They are still young.” (Participant 9)*

#### 3.1.3. Smaller Radius of Daily Activities

The participants reported that they did not go to places they usually went because the COVID-19 pandemic reduced their radius of daily activity. They did not go out unless it was absolutely necessary and tried to follow quarantine guidelines.

*“I used to go to department stores often, but I never go there anymore… I almost never go to large supermarkets anymore either.” (Participant 8)*

*“I would avoid going outside… I would not go out of the house unless it’s really necessary.” (Participant 1)*

#### 3.1.4. Changes in Exercise Patterns

The participants switched their exercises to indoor workouts such as squats or stretches. They also changed the time of day during which they exercised when working out outside of the house in order to exercise at a time of day when there was less traffic and adjusted their methods of exercise to be suitable for working out alone.

*“I do squats at home [for exercise].” (Participant 1)*

*“I take walks outside when there are fewer people. Early in the morning…” (Participant 2)*

*“I would avoid times when there are a lot of people. I exercise early in the morning.” (Participant 4)*

#### 3.1.5. Changes in Dietary Habits

In terms of dietary habits, the participants cooked at home or used delivery food services rather than eating out.

*“Eating out…I used to do it often. Before [COVID-19]… I still eat out, but less than half as often.” (Participant 13)*

*“Now I can’t go to chain supermarkets… I just buy food from neighborhood stores or get food delivered. Can’t go to restaurants…” (Participant 9)*

### 3.2. Increased Cautiousness in Daily Life

#### 3.2.1. Increased Caution toward Others

Most of the study participants said they were cautious toward others due to their fear of contracting COVID-19 and that they were hesitant to meet people. They expressed that this led to a decrease in face-to-face contact with people around them and led to a decrease in social communication overall. They avoided contact with people in confined spaces such as elevators and buses and stayed away from crowded places.

*“We can’t see who is infected. That’s why contact with other people is very scary.” (Participant 13)*

*“I avoid [people]. We need to avoid each other.” (Participant 1)*

*“I usually take the bus, so I am more concerned about contact with other people.” (Participant 8)*

#### 3.2.2. Avoiding Objects Interacted with by Others

Most of the participants reported that they avoided touching objects that other people had interacted with, just as they avoided strangers due to fears related to contracting COVID-19. They avoided using objects that many people touched, such as handles in buses or subway trains, as well as delivery packages, and cleaned objects touched by others and washed their hands after touching them.

*“I would not wear indoor slippers [at the hospital] because many people wear them.” (Participant 3)*

*“I would hold the handles [in the bus] with gloves on and use hand sanitizer before getting off.” (Participant 8)*

*“I would wipe [delivery boxes] intensely… I need to clean everything that goes in the fridge. I am stressed by cleaning those packages and putting them away.” (Participant 12)*

*“I would not like to exercise because I don’t want to touch the [public equipment]. I’m scared. People from here and there come and hold them and whatnot…” (Participant 2)*

### 3.3. Psychological Changes

#### 3.3.1. Boredom and Depression

Most participants said that their lives were not as fun and that they felt bored and depressed from being home alone since they could not meet friends and have conversations.

*“I felt helpless. It just happened.” (Participant 5)*

*“It takes effort to meet people and find friends but I can’t, so I feel depressed. I can’t go anywhere, like how I wish to…” (Participant 12)*

*“My life has simply become not as fun because of COVID-19.” (Participant 9)*

*“I almost died when the COVID-19 situation was severe because I couldn’t work and stayed home… I sometimes feel bored.” (Participant 7)*

#### 3.3.2. Fear of Infection

The participants expressed that they were bored and depressed due to being unable to meet people, but at the same time, they feared being infected with COVID-19. They also worried about their family members who had to interact with many people at their jobs.

They avoided going outside or participating in exercises such as hiking due to such fears. They also became sensitive to respiratory symptoms such as coughing and a sore throat and would sometimes visit the doctor as soon as these symptoms began.

*“I am afraid a lot of the time because the number [of infections] escalated.” (Participant 3)*

*“My daughter runs a hair salon, and COVID-19 makes me worried in many ways.” (Participant 9)*

*“This should subside soon. It scares me. It would not just be me if I get [COVID-19]. We have a lot of children. That’s why I am always cautious.” (Participant 6)*

*“It has become so rude to cough in public around others these days. It makes me flinch.” (Participant 6)*

#### 3.3.3. Increased Anxiety Caused by Media and Text Alerts

The participants felt anxiety in response to COVID-19-related news in media and daily text notifications updating them with the number of cases, which made them feel stressed due to excessive information.

*“I receive COVID-19-related texts every day. I can’t be relaxed when I see those. I am worried because it can be me at any time.” (Participant 9)*

*“I am worried because the number [of COVID-19 cases] goes down and up again.” (Participant 5)*

*“I almost feel neurotic. I always see it [on TV]… The texts come in—‘ding-dong’—every day.” (Participant 6)*

#### 3.3.4. Anger toward Others Who Do Not Follow Quarantine Rules

Meanwhile, the participants were angry when they saw others via the media or around them who went out and about and did not follow COVID-19 guidelines, and thought that those who did not wear masks were inconsiderate of others.

*“I feel angry when I hear the news that people go out and about and go on vacations.” (Participant 6)*

*“When I see five to six people gathering in groups without masks drinking and enjoying themselves, I feel that’s not right.” (Participant 11)*

### 3.4. Adaptation to Life during the COVID-19 Pandemic

#### 3.4.1. Enduring and Coming to Terms with the COVID-19 Pandemic

The participants acknowledged that COVID-19-related restrictions were necessary and willingly adjusted their lifestyles, feeling that it was necessary to endure the situation even if it caused inconveniences in daily life.

*“I would love to exercise, but following COVID-19 guidelines comes first. It’s not only me, but others are also enduring it, so we should do so as well.” (Participant 5)*

*“It’s really difficult wearing a mask itself. It’s hard because it gets so warm when I work with a mask on. Seriously… I get blisters all over.” (Participant 9)*

#### 3.4.2. Becoming Used to Life during the COVID-19 Pandemic

The participants adapted to the COVID-19 pandemic despite the hardships it brought. They said they became used to communicating with their families and friends via calls and text messages in order to follow COVID-19 guidelines, even though they were not able to meet in person.

*“Now, I am really getting used to it. At first it was frustrating and driving me crazy, but as we stayed like this for a long time, I think I’m really adapting to it.” (Participant 8)*

*“It’s much better even after a call. I feel like we are thinking about each other… It’s still incomparable to meeting in person.” (Participant 11)*

*“It used to be difficult and bothersome [to wear a mask] and I often forgot to do it… Now I look for the mask first thing in the morning and don’t even realize that I am wearing a mask all day.” (Participant 11)*

*“I think it has just become a part of daily life now. It was uncomfortable at first. I felt stuffy as I work, but now I’m just fine since I got used to it.” (Participant 8)*

## 4. Discussion

This is a qualitative study that examined the lifestyle changes of community-dwelling older adults with chronic diseases during the COVID-19 pandemic and the impact the pandemic had on their daily lives. The results of the analysis, which used the content analysis method, showed four general themes: “lifestyle changes,” “increased cautiousness in daily life,” “psychological changes,” and “adaptation to life during the COVID-19 pandemic.”

Since the first confirmed case of COVID-19 occurred in South Korea on 20 January 2020, COVID-19 prevention rules and instructions have been distributed, and social distancing has been maintained until now [31]. The unprecedented restrictions on individual activities as an effort to curb community spread of COVID-19 have significantly changed the lifestyles of older adults [20]. There have been several viral outbreaks in the recent past, including severe acute respiratory syndrome (SARS) in 2003 and Middle East respiratory syndrome (MERS) in 2012, but none of them persisted as long as the COVID-19 pandemic [32]. Even during the MERS outbreak in Korea in 2015, there was not a high level of social distancing or vaccine development, as infection spread through community transmission was rare, except for infections between family members or at medical facilities [33]. In contrast, the spread of COVID-19 was very fast, and it continues to spread as a serious new infectious disease that poses a global threat, with outbreaks in more than 200 countries, including China, Korea, Iran, Western European nations, and the United States [33]. A full year into the COVID-19 pandemic, many parts of people’s daily lives have changed [21]. Various media and news outlets reported exponentially increasing numbers of cases each day, and governments instructed people to stay in their homes and away from others, releasing measures and guidelines to prevent person-to-person spread of COVID-19 [34]. People had to minimize in-person meetings and gatherings, including get-togethers with family who lived separately, under circumstances in which they were told to practice social distancing, avoid gatherings of five or more people, and maintain personal cleanliness [18]. The small joys of life, such as meeting with friends, traveling, religious activities, and attending educational day programs at senior centers, stopped altogether, and sometimes the participants experienced psychological stress when they were required to quarantine after coming into contact with confirmed cases of COVID-19. Since older adults are more vulnerable to the negative impacts of COVID-19 than younger adults [20], policy-makers should also consider the negative results of social distancing and provide policy support to mitigate them. Wearing masks has been shown to be as effective at curbing the spread of the virus as social distancing [35]. The participants understood the importance of wearing masks and grew used to wearing them, even though they found masks uncomfortable when they were first required to wear them at work. Older adults made special efforts to practice hand sanitization at the individual level, which included washing hands and using hand sanitizer.

Older adults spent significantly more time at home than they did before the pandemic due to strict social distancing measures and the shrinking of their radius of daily activity. They expressed that they spent most of the time watching TV in order to avoid in-person contact and more time looking after their grandchildren who were not going to school during the COVID-19 pandemic. They avoided shopping at crowded places such as large supermarket chains and saw their mobility drop considerably due to restrictions regarding religious activities such as attending church. Increased time spent at home by older adults in this context can lead to many additional health issues [21]. It is well-known that a sedentary lifestyle has negative effects physically and mentally, such as a decrease in muscle strength and cognitive function and an increase in one’s sense of depression and isolation [36], as well as other health issues such as a vitamin D deficiency caused by insufficient exposure to sunlight [37]. Social isolation may cause immune system dysfunctions, increased susceptibility to infection, and aggravated pathophysiology [19]. Such negative health impacts from staying home may also lead to an increased risk of falling, which may cause injuries such as fractures or various other comorbid conditions, a significant factor influencing mortality among older adults [21].

Exercise patterns also changed. The participants lost the capability to go places where they used to exercise, including gyms, group exercise facilities, and community facilities, due to shutdowns and had to change their exercise patterns [18]. The participants in this study preferred indoor exercises such as stretching and squats, as they could not take part in in-person activities or group activities, and practiced walking through the corridors in their residential units or exercised outdoors early in the morning to avoid crowds. One’s individual participation level in exercise is determined by multiple factors, including environmental, physical, social, and political factors that influence one’s community [18,38]. Policy-makers should consider how the restricted physical environment for exercising and the social restrictions on group activities may negatively affect the exercise patterns of older adults. The 2020 study by Kwon suggested that older adults’ interest in exercising naturally subsided as they engaged in fewer activities and stayed home for an extended period of time due to social distancing [18]. Losing one’s exercise partner or having to exercise alone makes one more likely to give up exercising altogether or to become less committed to exercising, even if one recognizes the need to stay active [18,39,40]. A change in one’s exercise patterns to become more passive or give up exercise altogether likely leads to the weakening of one’s skeletal muscles and a decrease in one’s critical neuromuscular abilities [41]. Given that exercise is one of the most important factors for one’s health, bringing many health benefits and having a significant influence on healthy aging and quality of life [42,43,44], various programs to encourage seniors to stay active at home during the COVID-19 pandemic should be implemented.

The COVID-19 pandemic also had a significant impact on the dietary habits of older adults. Before the COVID-19 pandemic, participants who lived alone or with physical constraints were largely dependent on cafeterias in senior centers or soup kitchens as sources of their meals, and they struggled to prepare meals after these programs shut down due to the COVID-19 pandemic. Senior welfare centers have provided ready meal boxes for lunch and substitute instant foods, but these options cannot fully replace the meals that used to be offered by cafeterias. The participants who were able to function independently also avoided grocery shopping out of fear of contracting COVID-19 [45], and those who had difficulty preparing their meals due to health-related reasons skipped meals or replaced proper meals with instant food. In addition, eating alone, decreased physical activity, and anxiety and stress about possible COVID-19 infection can also lead to a decrease in one’s appetite, which can have an adverse effect on dietary health [20]. It is believed that a new welfare program is needed to identify and aid vulnerable older adults who are in a blind spot in the existing welfare system so that these older adults can maintain healthy eating habits even under circumstances of social distancing.

The participants said that they were increasingly cautious toward strangers in many aspects of daily life since the initial COVID-19 outbreak and were concerned about going to public or crowded places, meeting people, holding handles on the bus or the subway, and even about becoming infected from delivery boxes. They took extra efforts to protect themselves from the virus since older adults have a higher death rate from COVID-19 [4]. The participants avoided meeting friends or acquaintances and were hesitant to meet any people. They used gloves or hand sanitizer when touching objects in public places that were interacted with by others. Overall, the participants practiced social distancing and stayed at home in the COVID-19 era to protect themselves from the invisible virus.

The paradox of the key strategies of isolation and quarantine used to curb the spread of COVID-19 is that it causes new physical and mental health issues for older adults [3]. Older adults living in communities in this study expressed general disinterest, boredom, and depression due to prolonged social distancing and stay-at-home measures. Feelings of helplessness, loneliness, and depression comprise the psychological changes experienced by seniors during the COVID-19 pandemic as they spent extended periods of time at home alone without meeting with friends and family [1,46]. Despite the feelings of disinterest, boredom, and depression, older adults were also fearful of contracting COVID-19 and followed quarantine measures no matter how difficult it was for them. They followed the guidelines as much as they could, since being infected with COVID-19 would affect not only themselves, but also their loved ones.

At the same time, the participants in this study also reported anxiety due to the daily overflow of COVID-19-related news from media outlets and text alerts. News of COVID-19 cases reinforced anxiety among older adults and caused additional stress due to exposure to excessive information about COVID-19. They also showed anger toward people who did not follow the guidelines while they endured the difficult task of adhering to the guidelines. They thought that those who did not wear masks or who went on vacations were not considerate of others at a time when everybody should collectively follow the guidelines to stop the spread of COVID-19. Fear, stress, and loneliness caused by social isolation during the COVID-19 pandemic may aggravate the health of older adults [9]. Special attention should be paid to the health of older adults during the COVID-19 pandemic, given the physical and psychological problems caused by restricted mobility [3,13]. Due to limited community resources available during the COVID-19 pandemic [5], non-face-to-face ways to support older adults’ health should be developed in the non-contact era [12].

Despite various lifestyle changes, older adults adapted to the COVID-19 era in various ways. They overcame the initial inconvenience of wearing masks at the beginning of the pandemic and psycho-socially adapted to being unable to meet friends and family by replacing in-person communication with social media or phone calls. They recognized that they should prioritize social distancing above inconvenient changes in exercise or dietary habits in order to bring an end to the pandemic, and they also overcame their initial depression and anger about the pandemic, gradually accepting and adapting to the reality. This may be a component of people’s ability (i.e., resilience) to recover in the context of an infectious disease outbreak [15]. Another study on psychological factors related to COVID-19 lockdown adaptation conducted in Spain found that age was a key factor in the level of adaptation and that older adults adapted better to the COVID-19 lockdown [47]. Moreover, older adults’ wisdom and insight resulting from their diverse life experiences could have contributed to their high resilience, which can be a key positive factor for adapting to adversity [47].

This study found that older adults experienced various restrictions and lifestyle changes due to COVID-19 but gradually showed psychological adaptation to reduced social activities, wearing masks, and practicing hand hygiene. Nonetheless, there are limitations to this study. First, the interpretation of the results should take into consideration that this study targeted only South Korean older adults and that the experiences of older adults during the COVID-19 pandemic may vary according to the state of the spread of the virus, quarantine measures, and the socio-cultural environments of different countries. Second, this study was a cross-sectional study conducted at the 1-year point after the beginning of the COVID-19 pandemic. We suggest conducting a longitudinal study focused on psychological adaptation to lifestyle changes and adaptation to the COVID-19 pandemic through regular study. Future studies can use a mixed methods research design, such as the Questionnaire for Assessing the Impact of the COVID-19 Pandemic on Older Adults (QAICPOA) tool [48], for data collection and analysis to overcome the limitations of using a single research method.

## 5. Conclusions

This study provides a comprehensive and in-depth understanding of the changes in the lives of community-dwelling older adults with chronic diseases at the 1-year point since the initial outbreak of COVID-19 in South Korea. As a high-risk group, older adults showed increased caution in their daily lives due to fear of infection and experienced lifestyle and psychological changes due to the increased time spent at home and the decrease in social interactions. The participants in this study learned to accept the situation and tried to adapt as their social support systems were shut down and they increasingly spent time alone. Community-dwelling older adults with chronic diseases tried to adapt to the quarantine guidelines even when the social services network was locked down due to COVID-19. In situations where accessing face-to-face services is difficult, efforts should be made to improve overall quality of life for older adults with chronic diseases by developing and utilizing non-face-to-face support programs. Older adults with chronic diseases need community attention, as they are susceptible to the negative effects of restricted physical activity and psychological changes during the pandemic.

## Figures and Tables

**Table 1:** General characteristics of participants.

ID	Sex	Age	Education	Residence	Household Members	Number of Chronic Diseases	Occupation	Subjective Socioeconomic Status	Religion
1	Male	73	High school	Seoul	Married son	2	Part-time job	Middle	Catholic
2	Female	75	None	Seoul	Alone	7	None	Low	None
3	Female	80	Elementary school	Seoul	Alone	5	Part-time job	Low	None
4	Female	69	High school	Seoul	Alone	2	None	Low	Protestant
5	Female	80	High school	Seoul	Spouse	2	Part-time job	Low	Buddhist
6	Female	76	None	Seoul	Alone	3	Part-time job	Low	None
7	Female	74	Elementary school	Seoul	Alone	1	Part-time job	Middle	None
8	Female	66	High school	Daejeon	Spouse	1	Sanitation worker	Low	None
9	Female	65	Elementary school	Daejeon	Married daughter	3	Sanitation worker	Low	None
10	Female	78	Elementary school	Daejeon	Alone	1	None	Low	None
11	Male	69	Middle school	Seoul	Spouse	3	Part-time job	Low	Protestant
12	Female	77	High school	Gyeryong-si	Married son	3	Part-time job	Low	Buddhist
13	Male	76	High school	Gyeryong-si	Unmarried child	1	Part-time job	Middle	None

**Table 2:** Outcome of qualitative analysis.

Theme	Subtheme
Lifestyle changes	Following quarantine rules in daily life Increased time spent at home Smaller radius of daily activities Changes in exercise patterns Changes in dietary habits
Increased cautiousness in daily life	Increased caution toward others Avoiding objects interacted with by others
Psychological changes	Boredom and depression Fear of infection Increased anxiety caused by media and text alerts Anger toward others who do not follow quarantine rules
Adaptation to life during the COVID-19 pandemic	Enduring and coming to terms with the COVID-19 pandemic Becoming used to life during the COVID-19 pandemic

## Data Availability

The data presented in this study are available on request from the corresponding author. The data are not publicly available due to privacy or ethical restrictions.
